# Multi-sequence non-contrast MRI characterization of deep vein thrombosis in man

**DOI:** 10.1186/1532-429X-17-S1-P10

**Published:** 2015-02-03

**Authors:** Alkystis Phinikaridou, Prakash Saha, Marcelo E Andia, Alberto Smith, Rene Botnar

**Affiliations:** 1 Biomedical Egineering, King's College London, London, UK; 2 Academic Surgery, King's College London, London, UK; 3 Radiology, P, Santiago, Chile

## Background

Deep vein thrombosis (DVT) remains an important medical condition. The biophysical characteristics of thrombus may determine the response to endovascular interventions. We demonstrated that multi-sequence thrombus imaging (MSTI) using magnetization transfer rate (MTR), apparent diffusion coefficient (ADC) and T1 mapping can characterize thrombus organization and identify thrombi amenable to thrombolysis in a murine model. Here, we investigate whether MSTI can be translated to man and how these measurements associate with the outcome of intervention.

## Methods

MSTI was performed in patients with ilio-femoral DVT undergoing lysis at 3T using a 32-channel coil. T2-prepared, bSSFP MR venography (MRV) was acquired with: TR/TE=4.2/2.1ms, flip angle=700, FOV=220x299x200mm, matrix=112x148, slice thickness=2mm, resolution=2x2mm, averages=1, T2-prep-echo-time=30ms. 3D T1-weighted spoiled-GRE images were acquired with and without an on-resonance MT pre-pulse with: TR/TE=69/2.2ms, flip angle=180, FOV=220x299x198mm, matrix=112x148, slice thickness=6mm, resolution=2x2mm, averages=1. The binomial-block MT pre-pulse had a duration=1.92ms and repetitions=1. 2D diffusion weighted spin-echo images were acquired with: TR/TE=1780/82ms, flip angle=900, diffusion-echo-time=333ms, FOV=220x299x125mm, matrix=112x148, slice thickness=10mm, resolution=2x2mm, averages=2 and b-values=0, 333, 667, 1000 mm^2^/s. A 2D MOdified Look-Locker Inversion Recovery (MOLLI; 3-3-5) sequence was used for T1 mapping: TR/TE=3.3/1.6ms, flip angle=35°. FOV=220x299x198mm, matrix=112x148, slice thickness=6mm, resolution=2x2mm, averages=1. Thrombi were segmented on all images using Osirix and the T1, %MTR/cm^3^, and ADC values were reported.

## Results

**Figure 1:**
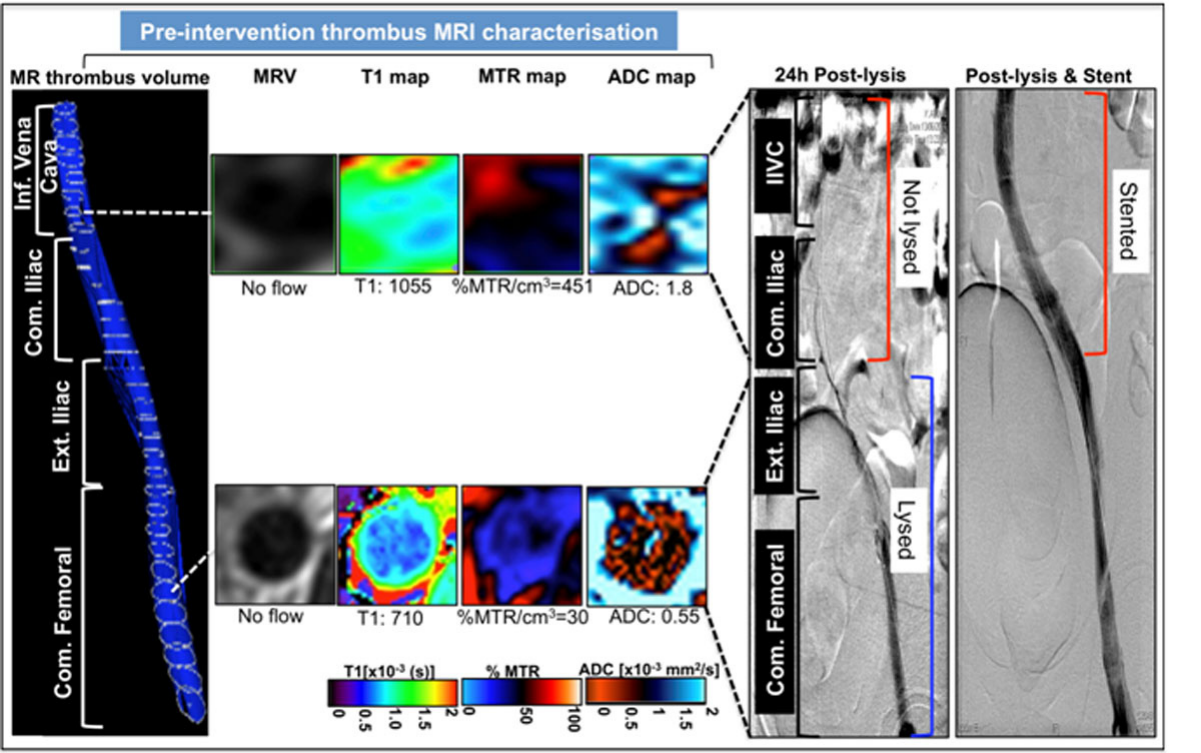
*In vivo* multi-sequence thrombus imaging

**Figure 2:**
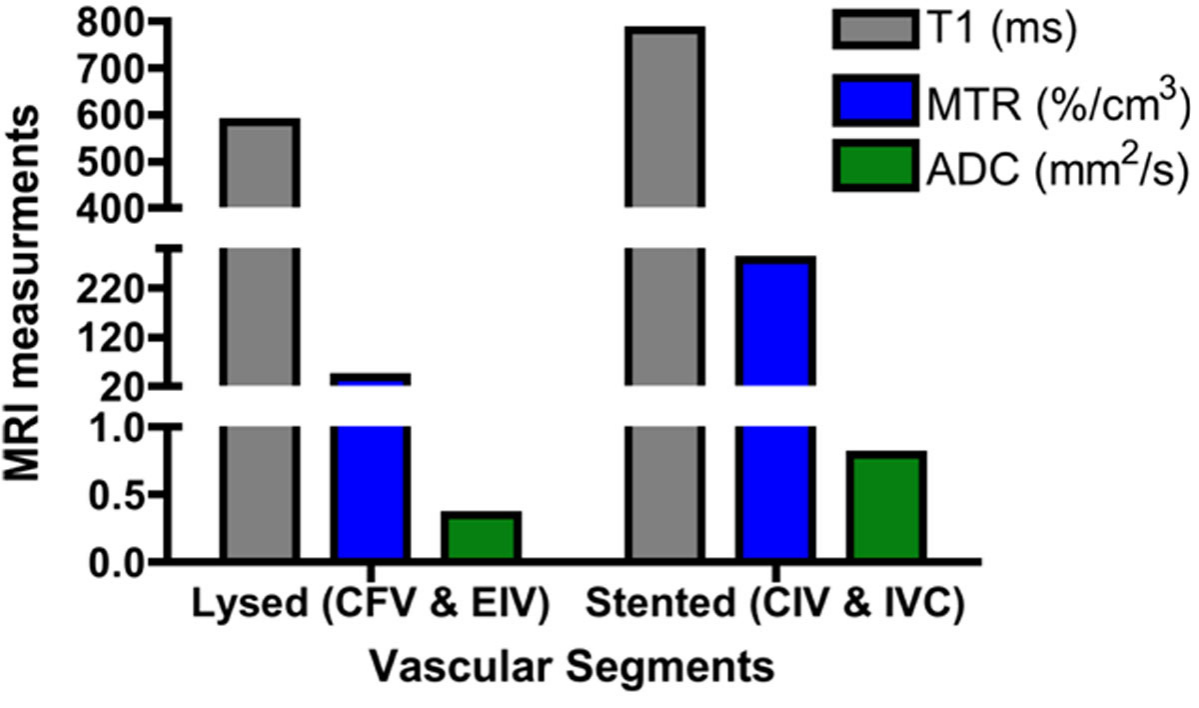
Quantitative MRI measurements differ between lysed and stented vascular segments

MSTI is feasible in man and successful characterization of ilio-femoral DVT was achieved in 30mins (Fig. 1). Figure 1A shows a volumetric reconstruction of a segmented thrombus that extended from the inferior vena cava (IVC) to the common femoral vein (CFV). Representative images of the segmented thrombus at 2 different levels revealed the structural heterogeneity within the same thrombus (Fig. 1B-1I). The thrombus in the IVC and CIV had a higher T1-relaxation time, MTR/cm^3^ and ADC values (Fig. 1C-E) compared to the thrombus in the external iliac (EIV) and common femoral (CFV) veins (Fig. 1F-I). Interestingly, the thrombus in the IVC and CIV did not lyse after 24h of thrombolysis, where as the thrombus in the EIV and CFV lysed, as seen on the invasive venogram (Fig. 1J). Recanalization of the thrombus in the IVC and CIV was achieved after stenting (Fig. 1K). Quantitative measurements of all the slices in each vascular segment are illustrated in Fig. 2.

## Conclusions

Non-contrast MSTI, using a combination of MTR, ADC and T1 mapping is feasible in man and may allow characterization of thrombus structure and understanding on how these measurements relate to the outcome of interventions.

## Funding

N/A.

